# Stochastic co-teaching for training neural networks with unknown levels of label noise

**DOI:** 10.1038/s41598-023-43864-7

**Published:** 2023-10-06

**Authors:** Bob D. de Vos, Gino E. Jansen, Ivana Išgum

**Affiliations:** 1 Department of Biomedical Engineering and Physics, Amsterdam University Medical Center, University of Amsterdam, Amsterdam, the Netherlands; 2 Department of Radiology and Nuclear Medicine, Amsterdam University Medical Center, University of Amsterdam, Amsterdam, the Netherlands; 3 Informatics Institute, University of Amsterdam, Amsterdam, the Netherlands

**Keywords:** Biomedical engineering, Computational science

## Abstract

Label noise hampers supervised training of neural networks. However, data without label noise is often infeasible to attain, especially for medical tasks. Attaining high-quality medical labels would require a pool of experts and their consensus reading, which would be extremely costly. Several methods have been proposed to mitigate the adverse effects of label noise during training. State-of-the-art methods use multiple networks that exploit different decision boundaries to identify label noise. Among the best performing methods is co-teaching. However, co-teaching comes with the requirement of knowing label noise a priori. Hence, we propose a co-teaching method that does not require any prior knowledge about the level of label noise. We introduce stochasticity to select or reject training instances. We have extensively evaluated the method on synthetic experiments with extreme label noise levels and applied it to real-world medical problems of ECG classification and cardiac MRI segmentation. Results show that the approach is robust to its hyperparameter choice and applies to various classification tasks with unknown levels of label noise.

## Introduction

Label noise in training data is detrimental to supervised training of deep neural networks^1,2^. Although training data without label noise is desired for accurate training, it is often impractical or impossible to attain. Especially in a medical setting, attaining high-quality labels with minimal errors would require a pool of experts labeling the data and, subsequently, their consensus readings to address disagreements, which is impractical and extremely costly. Matters are exacerbated by deep neural networks requiring large and diverse sets of training data. Consequently, overcoming the limitations of label noise when training neural networks is an active area of research^3–12^.

The definition of label noise may seem obvious: an instance, e.g., an image, is either correctly labeled or not. It may imply that the definition of label noise is always evident. However, class can be arbitrarily defined, especially in real-world medical settings. For example, medical diagnoses are often not dichotomous but follow a spectrum. As a consequence, borderline cases are difficult to label and are typically subject to observer bias. Furthermore, the definition of label noise becomes increasingly elusive for region-level classification and pixel-level classification. In such classification tasks, label noise not only refers to incorrectly labeled structures but may also refer to decisions subject to inter- and intra-observer variability. For example, in semantic segmentation, gross segmentations may be correct, but there is considerable variation in the definition of outlines of segmented structures, making label noise inherently present in semantic segmentation tasks.

Mitigating the negative impact of label noise in training deep neural networks can be achieved in multiple ways. For a comprehensive review of the methodology, we refer the reader to the paper by Song et al.^13^, and for an overview of applications in medical imaging we refer to Karimi et al.^14^. Briefly, methods focus on creating label noise invariant networks^15–18^, noise-tolerant loss-functions^19–23^, or on data-cleaning or re-weighting^12,24–39^. However, these methods either buckle under extreme amounts of label noise, require a subset of data free from label noise, or focus on a single classification task such as image-level instance-classification. Generally applicable state-of-the-art methods use multiple networks that are applied in sequence^40^ or in parallel^41–43^. They all rely on the principle that, although neural networks can memorize label noise, they will prioritize learning general patterns first ^1,2^.

Sequential models follow a paradigm related to knowledge distillation^44,45^. Jiang et al.^40^ proposed an approach where one network (MentorNet) is used to train another network (StudentNet). The goal of MentorNet is to learn a sample weighting scheme, i.e. a curriculum, that identifies correct and incorrect instances, and a StudentNet that learns the eventual task. The work is based on curriculum learning^46^, but unlike curriculum learning, it does not rely on a manually defined curriculum of easy and hard training instances, but it automatically defines one. However, the method relies on a small validation set of clean labels.

Methods that use networks trained in parallel focus on identifying label noise during training. Such methods are related to active learning^47^, boosting^48^, and bootstrapping^26^, but instead of employing one network, they use multiple networks that exploit different decision boundaries, i.e. epistemic uncertainty, to mitigate the negative effects of label noise, i.e., aleatoric uncertainty. For example, Malach et al.^41^ proposed a method that employs two networks and during training only uses instances to update the networks when they disagree. A similar approach was recently proposed where a voting scheme is used to select and reject samples from three networks^43^. However, these methods typically ignore the easy training instances, and may still include erroneously labeled training instances.

Han et al.^42^ overcome some of these limitations with co-teaching. In co-teaching, two models are independently trained using the same instances from a mini-batch but with a different selection. Each model selects training instances for the other model, dependent on the loss. Specifically, instances are evaluated per model, and a pre-specified amount of instances with the highest losses are rejected, i.e., “forgotten”, thereby selecting only the instances with the lowest losses for training the other model. An update to this technique was proposed by Yu et al.^49^ showing that a combination of previous techniques will keep the decision boundaries of the networks from converging to each-other during training^41,42^. Compared to conventional co-teaching, this approach achieved a marginal but consistent improvement in several classification experiments. However, a limitation of co-teaching is that a suboptimal forget-rate-parameter has a considerable negative impact on performance. Effective deployment of co-teaching requires a clean validation dataset, or at the very least knowledge about the amount of label noise. While this is not an issue in experiments where label noise is synthesized, it can be problematic in real-world tasks where a priori knowledge of label noise is hardly available and infeasible to attain.

We propose a method that introduces stochasticity to co-teaching for selection of correctly labeled and rejection of incorrectly labeled instances. Instead of using a predetermined forget-rate hyperparameter used in conventional co-teaching, we employ random selection thresholds on the posterior probability of training instances. This approach is loosely inspired by the effectiveness of random thresholds in extremely randomized trees^50^. With this approach we overcome the shortcomings of conventional co-teaching that hamper utilization. Similarly to regular co-teaching, stochastic co-teaching relies on two deep neural models that learn from each other. Two models with identical architectures are trained on the same data. However, given that these models are independently initialized and independently optimized, they will develop distinct decision boundaries. A stochastically determined threshold will be used to include or exclude an instance to update the other network, based on ground-truth-label posterior probabilities. The benefit of stochastic co-teaching is that it does not require assumptions on the expected amount of label noise.

We present several key-contributions in this paper. First, we propose a stochastically chosen posterior probability threshold for selecting and rejecting training instances for co-teaching. Second, we present an in-depth evaluation of the hyperparameters of this stochastic co-teaching and show the method is robust to varying degrees of label noise. Third, we show that stochastic co-teaching additionally provides an estimate of the amount of label noise. Fourth, we show superior performance of stochastic co-teaching on benchmark experiments using MNIST, CIFAR-10 and CIFAR-100 data. Finally, using multi-label ECG classification and semantic segmentation of cardiac MRI, we show that the method is generic and readily applicable to real-world medical tasks.

## Method: stochastic co-teaching

Co-teaching revolves around two models that are jointly trained whereby each model selects the instances to train the other model. Since each model is initialized differently, each model learns a different decision boundary, resulting in different selection of training instances. Conventional co-teaching^42^ depends on a predefined forget rate, that should be tuned towards the label-noise rate, to reject a fixed number of instances based on their largest losses. This severely limits its application to tasks where the amount of label noise is known beforehand. Instead, we propose a stochastic approach that does not reject a fixed number of instances. We exploit the posterior probabilities to select or reject training instances. Analogous to the lowest and highest losses used in conventional co-teaching, it is more likely for low-probability training instances to be labeled erroneously, than for instances with high posterior probability. However, it is unclear if the impact of this is more significant than other factors, such as low probabilities being associated with challenging (but correctly labeled) training instances. The question remains which threshold will optimally separate incorrect from correct instances. Since we assume to have no prior knowledge about the exact ground truth, and therefore no knowledge about the noise-rate either, we do not want to exclude all low-probability examples. Hence, we use a stochastic approach: instances are selected based on a threshold randomly chosen from a beta distribution. Algorithm 1 shows pseudo-code of our method.
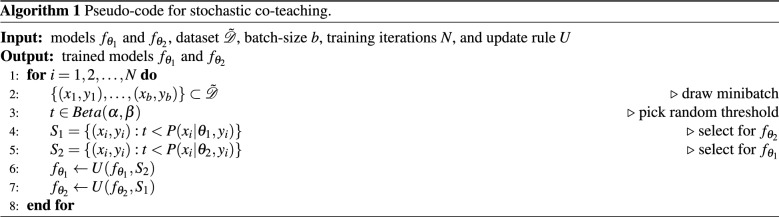


### Stochastic thresholds from a beta distribution

The thresholds that determine which instances are included are randomly chosen from a beta distribution. A beta distribution is defined on the interval $[0, 1]$:

1 $$\begin{aligned} Beta(\alpha ,\beta ): P(x|\alpha ,\beta )=\frac{x^{\alpha -1}(1-x)^{\beta -1}}{B(\alpha ,\beta )}, \end{aligned}$$

where *B* is the beta function

2 $$\begin{aligned} B(\alpha ,\beta )=\int _{0}^{1}t^{\alpha -1}(1-t)^{\beta -1} dt, \end{aligned}$$

which ensures a total probability of 1. The parameters $\alpha$ and $\beta$ determine the characteristics of the distribution and should be a non-zero positive number. Table 1 lists detailed properties of different values for $\alpha$ and $\beta$. In our experiments, we chose parameters $\alpha , \beta \ge 1$ such that the distribution is uniform or uni-modal. Note that increasing values of $\alpha$ and $\beta$ result in narrower distributions. Our experiments reveal that our approach benefits from $\alpha \gg \beta$, resulting in a narrow and left-tailed distribution. Thresholds randomly chosen from this distribution result in high values, but allow selecting lower thresholds, thereby offering chance to randomly select training instances with a low posterior probability that may represent difficult but not incorrect cases.

**Table 1:** Characteristics of the Beta-distribution. It is defined with parameters $\alpha , \beta > 0$ on the interval $[0, 1]$.

Parameters	Characteristics
$\alpha = \beta$	Symmetric
$\alpha , \beta < 1$	Bi-modal
$\alpha , \beta > 1$	Uni-modal, bell-shaped
$\alpha = \beta = 1$	Uniform
$\alpha < \beta$	Positively skewed
$\alpha > \beta$	Negatively skewed
$\alpha < 1, \beta \ge 1$	mode at 0
$$\alpha = 1, \beta > 1$$	
$\alpha \ge 1, \beta < 1$	mode at 1
$$\alpha > 1, \beta = 1$$	

### Numerical stability

The benefit of selecting and rejecting an arbitrary number of training instances from mini-batches may come at the cost of numerical instability. If all instances from a mini-batch would be rejected, an empty training set would be generated, which would cause numerical instability. To combat this, we employ two procedures. First, we clamp the randomly selected thresholds between 0.01 and 0.99. This ensures rejection of cases where the posterior probability is very low and it ensures selection of cases where the posterior probability is very high. Note that a threshold of 0 would result in inclusion of all instances and a threshold of 1 would result in rejection of all instances and thus an empty set. Second, we monitor the fraction of selected training instances and impose the following selection criterion: when $<10\%$ of the instances in a mini-batch are selected, we generate a new selection threshold. When five consecutive thresholds do not satisfy the selection criterion, the mini-batch may exclusively consist of instances with label noise, and a new mini-batch is sampled.

Similarly to^42^, we gradually introduce stochastic co-teaching via a schedule. The schedule introduces the selection threshold gradually

$$\begin{aligned} Beta(\alpha , \beta )\cdot \eta _n \end{aligned}$$

with multiplication factor

$$\begin{aligned} \eta _n = \max (0, \min (1, (n-n_0)/\delta )), \end{aligned}$$

where $n$ is the current time-step, and $n_0$ the time-delay to start the introduction gradually in $\delta$ steps.

### Rejection rate

In contrast to conventional co-teaching, stochastic co-teaching can reject an arbitrary number of training instances. By calculating the rejection-rate (per epoch) training stability can be monitored. Instability appears when the majority of instances is rejected during training. Furthermore, assuming that only training instances with incorrect labels are rejected, the rejection-rate provides an estimate of label noise. Note that the rejection-rate is equivalent to the term forget-rate used in conventional co-teaching, but for clarity we use the two terms distinctly.

## Experiments and results

**Figure 1:**
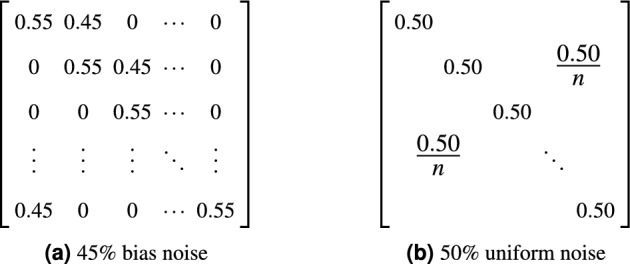
Noise transition matrices used in synthetic experiments. Transition matrices are equivalent to confusion matrices but they visualize noise distribution among classes. ias noise, or label-flipping noise, mimics observer-bias by substituting the true label with the label of the neighboring class. Uniform noise is achieved by replacing the true label with a randomly selected other label. Note that bias noise should always be <50% to ensure that the majority of instances remain correct.

For a comparison with previously published methods we have performed baseline experiments with synthesized label noise in MNIST^51^, CIFAR-10^52^, and CIFAR-100^53^ datasets. To generate label noise we employed two types of noise transition matrices visualized in Fig. 1. Bias noise, also referred to as label-flipping noise^42,49^, mimics observer-bias by substituting a ground-truth label with the label of the neighboring class. Uniform noise is achieved by replacing a ground-truth label with randomly selected other label. For a direct comparison with related methods, experiments were performed similar to those in^17,26,42,54^, i.e. experiments with high levels of label noise: bias noise with a noise rate of 45%, and uniform noise with noise rates of 50% and 20%. Note that experiments with 45% *bias* noise are exceptionally difficult, because there is a marginal majority of correct samples; a noise rate of more than 50% bias noise would flip the majority of instances to the incorrect label.

We implemented two CNN architectures, a standard four-layer CNN for MNIST experiments, and a nine-layer CNN for CIFAR experiments. The former model was specifically designed for MNIST and highly efficient and fast and the latter has been used for experiments with weak supervision and noisy labels^42,55^ being less efficient and therefore slower. Table 2 lists the architectures of these networks.

Each network was trained in 200 epochs in mini-batches using stochastic gradient descent with Adam and a learning rate of 0.001. Regular co-teaching was performed using optimal settings reported in^42^. In each experiment, Stochastic Co-Teaching was introduced in ten epochs; in MNIST experiments without a delay and in CIFAR experiments with a delay of ten epochs. If not stated otherwise, the reported results and corresponding standard deviations are determined from the last ten epochs of each experiment.

All experiments were implemented using PyTorch^56^ and were performed in accordance with relevant guidelines and regulation.

**Table 2:** The two network architectures used in the experiments. The MNIST model uses ReLU as activation function. The CIFAR model, as used in^42,57^, uses batch-normalization after each convolution layer and uses a leaky-ReLU for activation with a negative slope of 0.01.

MNIST	
28x28 input	
32 * 3x3 conv.	
64 * 3x3 conv.	
2x2 max pooling	
0.25 dropout	
128 nodes	
128 nodes	
0.50 dropout	
n classes	

CIFAR10/CIFAR100	
32x32 input	
128 * 3x3 conv.	
128 * 3x3 conv.	
128 * 3x3 conv.	
2x2 max pooling	
0.25 dropout	
256 * 3x3 conv.	
256 * 3x3 conv.	
256 * 3x3 conv.	
2x2 max pooling	
0.25 dropout	
512 * 3x3 conv.	
256 * 3x3 conv.	
128 * 3x3 conv.	
average pooling	
n classes	

### Hyperparameter stability

**Figure 2:**
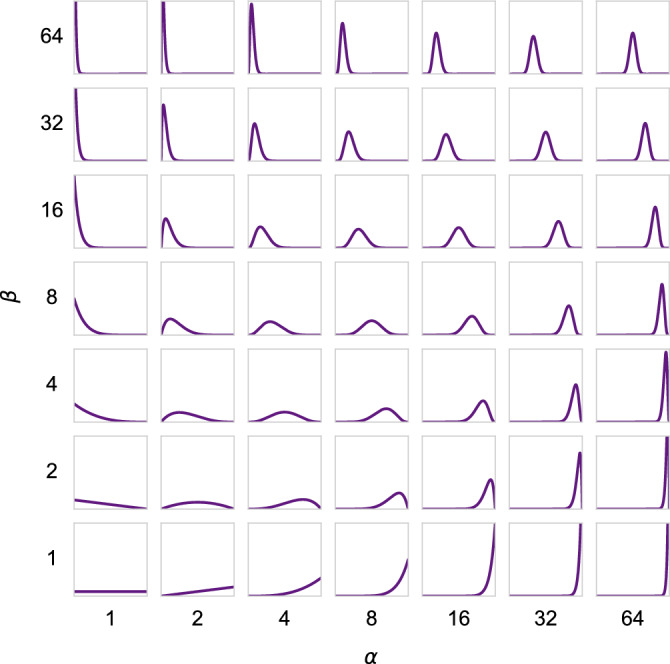
Variations for $\alpha$ and $\beta$ used in experiments result in varying Beta probability distributions.

We propose a stochastic co-teaching approach, where a randomly chosen threshold is used to select or reject training instances based on posterior label-probability. We have performed extensive experiments to investigate the influence of hyperparameters $\alpha$ and $\beta$, i.e. the hyperparameters that determine different beta distributions to sample the instance-selection thresholds. Figure 2 shows the $\alpha$ and $\beta$ parameters used in the experiments and their corresponding beta distributions. The probability densities show a wide variety of shapes, ranging from uniform, to parabolic, to bell-shaped distributions that are symmetric, or right- or left-tailed. Selection thresholds are stochastically sampled from these distributions and used in the experiments.

**Figure 3:**
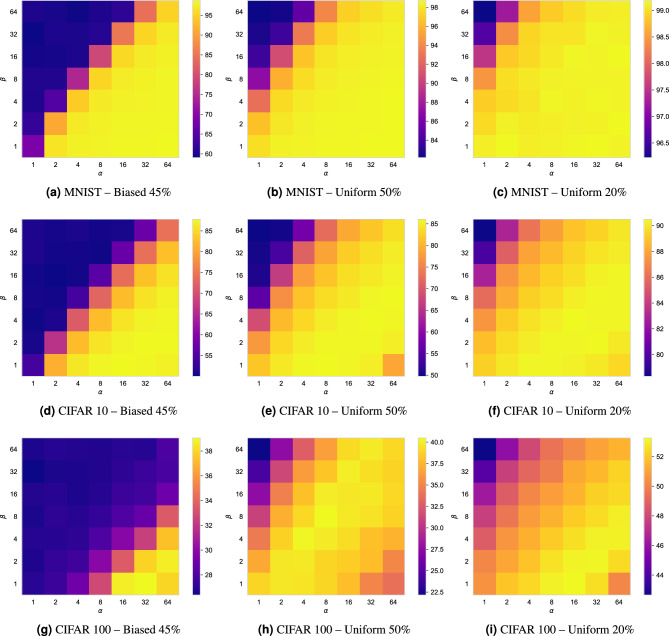
Hyperparameter-sweep experiments showing accuracy of the average of last 10 epochs of 200 epochs. Figures (a)-(i) show the experiments that were performed, using corresponding variations of Beta-distributions shown in Fig. 2. Each colorbar is scaled to the specific range of results.

Figure 3 shows the effect of different hyperparameters on stochastic co-teaching. The different beta distributions have different impact, but there are dominant hyperparameters pairs that achieve optimal results in nearly all experiments. In general, the distributions above the $\alpha = \beta$ diagonal (i.e. the right-tailed distributions) show suboptimal results and the distributions below the $\alpha = \beta$ diagonal (i.e. the left-tailed distributions) show optimal results. Right-tailed distributions sample lower thresholds on average, meaning an increased chance of selecting instances with low posterior probabilities. Left-tailed distributions sample higher thresholds on average, meaning an increased chance of rejecting instances with low posterior probabilities, leaving mainly high posterior probability instances.

Not all hyperparameters perform equally in all experiments. CIFAR-10 and CIFAR-100 image classification tasks are generally considered more challenging. In these applications, there are narrower sweet spots for hyperparameters. The left-tailed somewhat wider bell-shaped distributions appear to be optimal, with ${\alpha =32, \beta =2}$ as an optimum in all applications. Nevertheless, hyperparameters show relatively wide sweet spots, and different hyperparameters have limited impact on accuracy.

### Rejection rate

**Figure 4:**
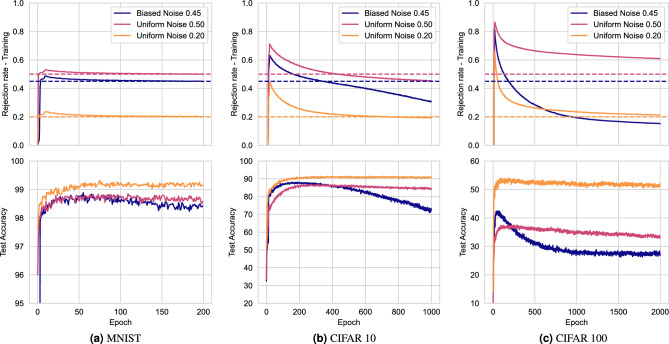
Stochastic co-teaching provides an estimate of label noise rates, if both the rejection rate and the test/validation performance converge. Rejection rates and test accuracy are shown for image classification in (**a**) MNIST, (**b**) CIFAR-10, and (**c**) CIFAR-100 classification experiments. We used Beta-distributions with parameters $\alpha = 32$ and $\beta = 2$. The real noise-rates are provided as a dashed horizontal line in matching colors.

Stochastic co-teaching can estimate the amount of label noise present in the data by monitoring the rejection rate. Figure 4 shows the development of the rejection rate during training in several applications. From the figure we observe that when the accuracy converges, the rejection rate converges towards the noise-rate. For CIFAR classification, the rejection rate overshoots for the more challenging tasks with higher noise rates. However, this faulty estimation can be inferred from deteriorating test or validation accuracy.

### Comparison with other methods

When the label noise-rate is known, conventional co-teaching outperforms competing methods on synthetic tasks, as was reported by Han et al.^42^. The results of our experiments are shown in Table 3. In all but one experiment, stochastic co-teaching outperforms its conventional counterpart. The benefit of stochastic co-teaching is that the method allowed the same parameter setting ($\alpha = 32$ and $\beta = 2$) for all experiments. In addition, the rejection rate of stochastic co-teaching provides insight about the label noise rate.

**Table 3:** Comparison of the accuracy achieved by stochastic co-teaching and previous methods. For a fair comparison we report the results from Han et al.^42^, as well as our replication of these experiments. We report the results of related methods Decoupling and MentorNet, and we report results of standardly trained neural networks (Standard), co-teaching (CoT), and stochastic co-teaching (StoCoT). For stochastic co-teaching we chose a left-tailed beta distribution with parameters $\alpha =32$ and $\beta =2$. Note that there is a performance increase in our CoT experiments in all but the CIFAR 100 experiments. Figure 5 shows that random initialization has an impact on an ill-tuned forget rate.

Dataset	Noise	Reported by Han et al.^42^				Our experiments		
		Standard	Decoupling	MentorNet	CoT	Standard	CoT	StoCoT
MNIST	bias 45%	$56.52 \pm 0.55$	$58.03 \pm 0.07$	$80.88 \pm 4.45$	$87.63 \pm 0.21$	$59.42 \pm 1.41$	$93.49 \pm 0.50$	**98.35** ± 0.09
MNIST	uniform 50%	$66.05 \pm 0.61$	$81.15 \pm 0.03$	$90.05 \pm 0.30$	$91.32 \pm 0.06$	$81.27 \pm 0.29$	$96.63 \pm 0.09$	**98.62** ± 0.06
MNIST	uniform 20%	$94.05 \pm 0.16$	$95.70 \pm 0.02$	$96.70 \pm 0.22$	$97.25 \pm 0.03$	$96.08 \pm 0.23$	$98.32 \pm 0.07$	**99.13** ± 0.08
CIFAR10	bias 45%	$49.50 \pm 0.42$	$48.80 \pm 0.04$	$58.14 \pm 0.38$	$72.62 \pm 0.15$	$50.56 \pm 0.86$	$77.97 \pm 0.38$	**87.69** ± 0.30
CIFAR10	uniform 50%	$48.87 \pm 0.52$	$51.49 \pm 0.08$	$71.10 \pm 0.48$	$74.02 \pm 0.04$	$50.17 \pm 0.99$	$81.65 \pm 0.19$	**85.79** ± 0.28
CIFAR10	uniform 20%	$76.25 \pm 0.28$	$80.44 \pm 0.05$	$80.76 \pm 0.36$	$82.32 \pm 0.07$	$77.72 \pm 0.48$	$87.72 \pm 0.18$	**90.46** ± 0.20
CIFAR100	bias 45%	$31.99 \pm 0.64$	$26.05 \pm 0.03$	$31.60 \pm 0.51$	$34.81 \pm 0.07$	$26.62 \pm 0.37$	$27.68 \pm 0.26$	**37.53** ± 0.43
CIFAR100	uniform 50%	$25.21 \pm 0.64$	$25.80 \pm 0.04$	$39.00 \pm 1.00$	**41.37** ± 0.08	$19.60 \pm 0.54$	$36.77 \pm 0.27$	$37.95 \pm 0.28$
CIFAR100	uniform 20%	$47.55 \pm 0.47$	$44.52 \pm 0.04$	$52.13 \pm 0.40$	**54.23** ± 0.08	$38.86 \pm 0.52$	$47.99 \pm 0.21$	$53.16 \pm 0.26$

In the replication of conventional co-teaching experiments, we achieved higher accuracies than reported by Han et al.^42^. To inspect the cause of this we studied the effect of the forget-rate hyperparameter and the effect of different random seeds used for parameter initialization and mini-batch sampling. For each experiment, we used forget rates between 5% and 95% in steps of 5%. We repeated each MNIST experiments ten times with different random initialization. For CIFAR experiments we repeated each experiment five times, because training this network was more time consuming (0.5 h vs. 3 h.).

**Figure 5:**
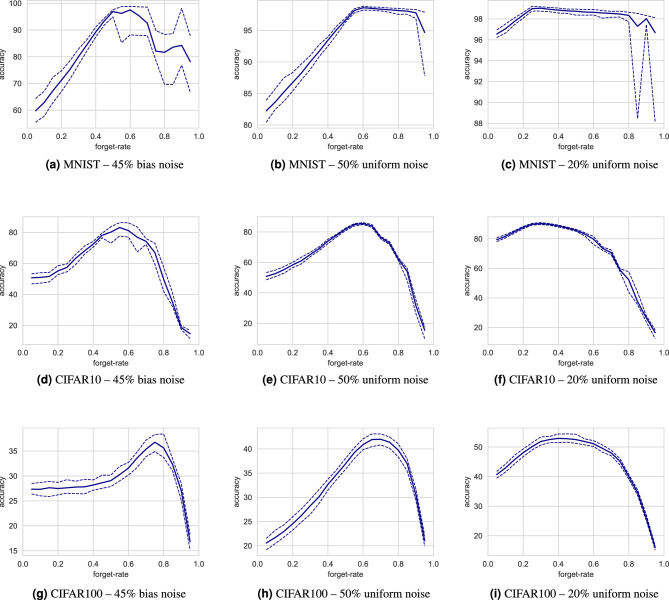
Accuracy of conventional co-teaching using different settings for the forget-rate hyperparameter. The solid line shows average accuracy and dashed lines show the range of accuracies, calculated for 10 experiments per forget rate for MNIST, and 5 experiments per forget-rate for CIFAR 10 and CIFAR 100. The results demonstrate the impact of an ill-chosen forget-rate and different random initializations of conventional co-teaching.

The results, shown in Fig. 5, reveal that conventional co-teaching is sensitive to its forget-rate hyperparameter. When the noise-rate is known and the forget-rate is chosen equal to it, a suboptimal accuracy is achieved, concurring with the findings reported by Han et al.^42^. Additionally, our results demonstrate that the impact of overestimating the forget-rate is larger than the impact of underestimating it, particularly for experiments with CIFAR-10 and CIFAR-100. Furthermore, different random seeds resulted in a large range of achieved accuracies, specifically in the experiments with MNIST data.

## Real-world medical tasks

To show applicability of our method to medical data we employed stochastic co-teaching for multi-label classification of medical signals, namely ECGs and for semantic segmentation of medical images, namely cardiac cine MRI. Like any other medical task, this data inherently contains label noise caused by, e.g., inter- and intra-observer variability.

### ECG classification

ECG is the primary tool for cardiologists to assess cardiac condition of patients. A typical ECG exam acquires 10 seconds of data at 500 Hz using 12 leads. ECG characteristics are sometimes automatically detected, but an ECG is thereafter manually assessed for diagnosis. This manual interpretation task can be cumbersome and it is often non-trivial in the presence of pathology^58^. Automatic interpretation of ECGs using deep neural networks is currently subject of intensive research, but training such networks is non-trivial, because ECG interpretation is complex due to label noise and observer bias.

In this experiment, we apply stochastic co-teaching to ECG classification using the PTB-XL dataset^59,60^. A full description of the data can be found in^59^. Briefly, the dataset consists of 21,837 clinically acquired 12-lead ECGs of 10 seconds (16 bit, 500 Hz) from 18,885 patients. The data are divided into ten folds of equal size on the patient-level. The 71 different features and diagnoses are aggregated into 5 different classes. The task is posed as non-exclusive multi-label classification in the following classes: normal, conduction disturbance, myocardial infarction, hypertrophy, and ST/T changes. We divide the folds into training, validation, and test data as proposed in^59^. Only some of the training data labels were scrutinized by an expert and therefore the training data contains label noise. The validation and test data were checked by an expert and can be considered to contain minimal levels of label noise.

**Figure 6:**
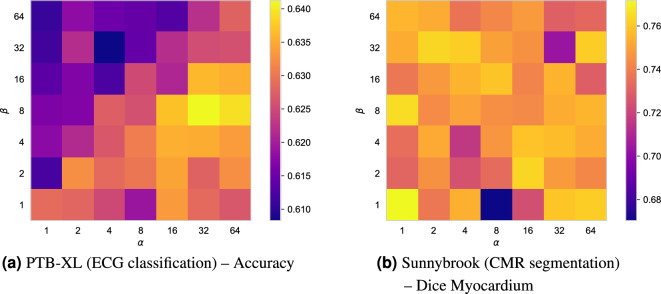
Performance of proposed stochastic co-teaching on real-world medical tasks for different hyperparameter settings of $\alpha$ (horizontal axis) and $\beta$ (vertical axis): (**a**) Accuracies for Multi-label ECG classification (PTB-XL). (**b**) Dice scores for left ventricle myocardium segmentation in cardiac MRI (CMR). The baseline ECG classification network trained without stochastic co-teaching achieved an accuracy of 0.618, and the baseline CMR segmentation network a Dice score of 0.71.

We have performed experiments using a Resnet adapted for time-series^61^, which is a top-scoring neural network on the PTB-XL data^60^. In Table 4, we present baseline results as reported in Strodthoff et al.^60^, our replication of the baseline method, and results of our proposed stochastic co-teaching. The results show similar AUCs between our implementation of the baseline method and stochastic co-teaching: 0.913 and 0.917, respectively, with overlapping confidence intervals. Note there is a difference between the results reported in Strodthoff et al. and our replication, likely caused by implementation differences. Our implementation of the trained baseline network achieved an accuracy of 0.618, with stochastic co-teaching the accuracy increases to >0.640 as is shown in Fig. 6a. Different hyperparameter settings show a similar pattern of performance as is shown in Fig. 3: higher performance is below the $\alpha = \beta$ diagonal.

**Table 4:** Macro-averaged AUCs for *super-diagnosis* (i.e. course-grained) classification of ECG from the the PTB-XL dataset. Following Strodthoff et al.^60^, we indicate the 95% confidence interval between brackets by bootstrapping the test set. The first row shows baseline results from Strodthoff et al.^60^, the second row shows our replication of the experiment, and the last row shows the result from the experiment with stochastic co-teaching. For Stochastic Co-Teaching, hyperparameter settings $\alpha =32$, and $\beta =2$ were used.

	AUC
Reported by Strodthoff et al.^60^ – Baseline	0.930 (0.005)
Our experiments – Baseline	0.913 (0.032)
StoCoT	0.917 (0.029)

### Cardiac MRI segmentation

In this experiment we evaluate stochastic co-teaching for left ventricle segmentation in cardiac cine MRI images. These images are typically acquired to evaluate cardiac function. One of the primary indicators of cardiac function is the ejection fraction. Ejection fraction is the fraction of blood that leaves the heart when it contracts. It is calculated from annotations of the endocardium (the inner wall) of the left-ventricle at two time-points: at end-diastole (maximum expansion) and end-systole (maximum contraction). While segmentation may seem trivial, the endocardium contains many papillary structures (i.e. protruding muscle tissue) that make the task prone to high intra- and inter-observer variability. Moreover, some of the papillary muscles are quite large and this may affect measurements if inconsistently segmented. However, segmenting papillary muscles is cumbersome.

In this experiment we use publicly available MRI images from the Sunnybrook challenge^62^. The dataset consists of short-axis cardiac cine MRIs from 45 patients. Multiple slices are acquired that encompass the heart. Image resolution is 1.$25 \times 1$.25 mm in-plane. Each slice is a time-series of 24 frames visualizing one heart-beat. For this dataset three structures are annotated at end-diastole and end-systole. In approximately half of the images, the two largest papillary muscles have been annotated as a separate class. We have included those in our experiments.

We divided images into a training set (104 images) and a test set (49 images) on the patient level such that the test set does not contain images from patients in the training set. We mimic segmentation errors by assigning papillary muscles to the blood pool in 40% of the image slices in the training set. We did not modify the test-set. We performed segmentation experiments with a U-Net^63^, because this is one of the most used architectures for medical image segmentation. The network was trained in 1000 epochs using mini-batches containing 16 randomly selected image patches of 128 × 128 pixels (original image size is 256 × 256). Other augmentations were random flipping and random rotations around all axes in steps of 90 degrees.

**Figure 7:**
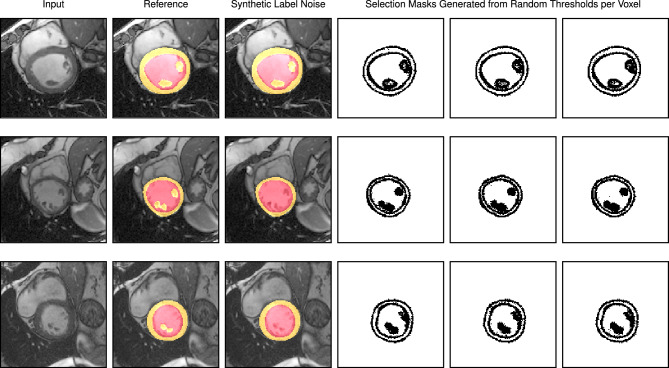
Three cardiac MRI training images (first column), with the original reference labels (second column), and with synthetic label noise applied (third column). The fourth to sixth columns show different selection masks generated by stochastic co-teaching. Left ventricle myocardium is indicated in yellow, and the blood pool in red. The selection masks indicate selection (white) or rejection (black) of pixels for training. Top row: A training example with annotated papillary muscles, hence no synthetic label noise applied. Note that pixels are rejected exclusively along segment borders, while the pixels in the center of the papillary muscles are selected. Middle row and bottom row: Two training examples with synthetic label noise. Synthetic label noise is added by including pixels representing papillary muscle in the blood pool. Note that for all selection maps in the middle and bottom row, pixels representing papillary muscles are rejected for training. These examples show that the method rejects the noisily labeled papillary muscles, while preventing the overfitting of correctly labeled papillary muscles in the first example. See Table 5 for quantitative results.

We implemented stochastic co-teaching to select or reject in individual voxels during training. By visualizing selection and rejection of voxels as masks, valuable qualitative information during training is revealed, such as areas with label errors and areas of observer variability, as is shown in Fig. 7. The selection masks ignore borders of segmentations. This is logical considering that the outlines can be quite arbitrary. Furthermore, the selection masks include inner voxels of papillary muscles when they are correctly labeled, and they exclude them when they are not labeled, meaning that stochastic co-teaching has effectively ignored incorrect labels. Note that a single threshold might be used to select pixel-instances in a mini-batch, but we chose to generate a selection threshold for each pixel. However, given that generating random parameters is time-consuming, we generate one 16 × 16-map per training image patch and tile these to the patch size.

**Figure 8:**
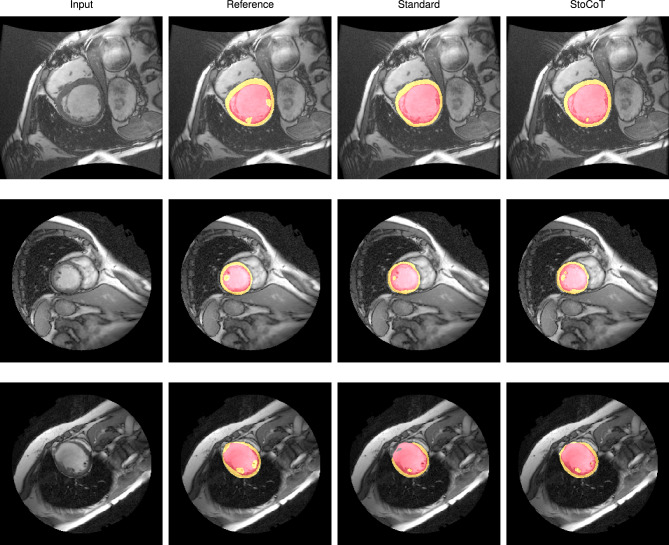
Examples showing the segmentation performance on the test-set of a standard CNN and the similar CNN with stochastic co-teaching. Co-teaching shows more sensitivity towards segmentation of papillary muscles, even though their segmentations were excluded in 45% of training data. See Table 5 for quantitative results.

Figure 8 shows several qualitative examples of a U-Net trained with and without stochastic co-teaching. The results show that the method using stochastic co-teaching achieves a more consistent output. The method more consistently segments the papillary muscles and the results also show that it outlines the myocardium more consistently. Quantitative results, listed in Table 5, demonstrate that Dice and distance metrics improve when stochastic co-teaching is applied compared to standard training. Finally, Fig. 6b demonstrates the robustness of the stochastic co-teaching towards this segmentation task with varying settings of hyperparameters $\alpha$ and $\beta$.

**Table 5:** Cardiac MRI segmentation of the left ventricle blood pool and myocardium. Compared with a standardly trained U-Net (Standard), stochastic co-teaching (StoCoT) results in higher Dice and lower distance metrics owing to its robustness against label noise. Stars indicate p-values determined by one-sided Wilcoxon signed-rank tests (* for $p \le 0.01$, and ** for $p \le 0.001$). See Fig. 5 for qualitative results.

	Blood Pool	Myocardium
(a) Dice coefficient		
Standard	0.89 (0.13)	0.71 (0.13)
StoCoT	0.91 (0.04)*	0.73 (0.09)
(b) Hausdorff distance		
Standard	9.05 (5.06)	15.62 (5.78)
StoCoT	7.46 (3.89)**	14.53 (3.76)
(c) Mean surface distance		
Standard	1.61 (1.13)	1.88 (1.45)
StoCoT	1.35 (0.45)**	1.59 (0.61)

## Discussion

Stochastic co-teaching employs two neural networks that are jointly trained. Each network selects mini-batch examples for the other using a stochastically determined threshold on the posterior probability. This approach does not require a priori knowledge about label noise, and as a result it eliminates the need for meticulous parameter tuning, which is especially useful in real-world tasks. Stochastic co-teaching achieves excellent results and outperforms state-of-the-art approaches on a variety of classification tasks with extreme and unknown levels of label noise. It is robust to varying levels of label noise and it can be used to estimate the level of label noise by monitoring the rejection rate. Furthermore, we have demonstrated the applicability of our method in two real-world medical tasks: classification of ECG signals, and semantic segmentation of cardiac MRI images.

Incorrect estimation of the noise rate may be detrimental to model performance. As Fig. 4 shows, the noise rate is incorrectly estimated for the uniform 50%, and biased 45% noise cases in the CIFAR experiments. This can be caused by the complexity of the problem, and by poorly chosen $\alpha$ and $\beta$. However, we have demonstrated that with a priori unknown noise rates, stochastic co-teaching requires relatively little hyperparameter tuning compared to conventional co-teaching. While conventional co-teaching requires extensive tuning of the forget rate, in stochastic co-teaching, the forget rate is automatically determined. The only hyperparameters for stochastic co-teaching pertain to the shape of the sampling distribution, defined by $\alpha$ and $\beta$. As the hyperparameter grids shown in Fig. 3 suggest, the performance is consistent for a wide range of settings and problems. Similarly, the results from cardiac MRI segmentation problem demonstrate relatively homogeneous performance across the different hyperparameter settings. The ECG classification results on the PTB-XL dataset show a slightly lower fault tolerance with respect to choosing $\alpha$ and $\beta$, but they agree with the general trend that it is beneficial to choose $\alpha > \beta$, as observed in MNIST and CIFAR. We note that other distributions could potentially be chosen for threshold sampling: the only requirement is that the distribution is defined exclusively on [0, 1], and that it is unimodal, which the beta distribution satisfies for $\alpha , \beta > 1$. Additionally, left-tailed distributions are preferred, like the $\beta$-distribution for $\alpha >\beta$. However, these results may be specific to experiments performed with softmax outputs and cross-entropy. Since different losses are differently calibrated^64^, they might show other responses to stochastic co-teaching.

Replication of baseline experiments with conventional co-teaching resulted in higher accuracies than reported by Han et al.^42^. Differences in results may be ascribed to differences in random initialization. Namely, in our experiments we have shown that different random initialization of conventional co-teaching resulted in highly variable outcomes. In cases of extreme label noise, mini-batches might be sampled consisting predominantly of training-instances with label noise. Such situations are difficult for conventional co-teaching where a predetermined number of training instances is selected. Stochastic co-teaching handles these situations better since it can reject an arbitrary amount of label noise. However, we have observed that mini-batches containing only instances with label noise led to numerical instability when all instances were rejected. We have addressed this by generating new selection thresholds or by resampling new mini-batches. The stochasticity has an additional effect of enforcing different decision boundaries to each of the models during training, and although we did not study the effects, we presume that stochastic co-teaching allows training of models that were initialized identically, meaning that stochastic co-teaching might be readily applicable to pre-trained networks.

Similar to conventional co-teaching, we employed a schedule that introduces the selection threshold of stochastic co-teaching. For complex tasks we found that a delay of a few several epochs benefited performance. This delay effectively utilizes the tendency of deep neural networks to learn general patterns first^1,2^. While we did not perform an in-depth study of the effect of different schedules on different classification tasks, network architectures, or loss functions, we observed a limited effect in preliminary experiments.

Stochastic co-teaching in the PTB-XL ECG classification task leads to improved accuracy, whereas the AUC remains similar to the baseline. This may indicate that the improvement from stochastic co-teaching is caused by improved classification of the majority classes. The difference between our baseline experiments on the PTB-XL ECG classification dataset and those from Strodthoff et al.^60^ may be attributed to differences in randomness-based operations, such as weight initialization and data sampling.

A drawback of co-teaching is that training with two networks imposes increased computational demands of approximately twice the amount of compute and memory compared to supervised training. A more efficient alternative is bootstrapping, which uses just one network for selection or rejection of samples^26^. Bootstrapping handles label noise well, but it is outperformed by co-teaching^42^. This indicates that it is beneficial to train with two predictors, each with its own decision boundaries. An alternative method including more than two networks was also proposed^43^. However, such an approach increasingly impacts hardware demands, especially for semantic segmentation tasks, which require high resolution outputs. Alternative directions might be in training Bayesian networks, and synthetically increasing the number of networks. However, each of the methods would require non-trivial voting schemes. Furthermore, consensus voting and selecting instances for training renders the method more similar to Decoupling^41^ where the error propagation is strongly coupled among networks^49^, contrasting with the core benefits of co-teaching where networks are decoupled and thus decision boundaries can develop individually.

Although we only evaluated stochastic co-teaching for classification tasks using the cross-entropy loss, other losses could be applied. However, threshold selection should be re-evaluated, because the effect of hyperparameters might be different than in our experiments. Additionally, co-teaching could be recasted for application in regression problems. The decision of selection and rejection should then be a distance metric, e.g. an L1 or L2 norm and the inclusion threshold should be sampled from an unbounded distribution, e.g. a Gamma or a Chi-squared distribution.

## Conclusion

We have presented a method for stochastic co-teaching. The method employs training of two networks where each network selects training instances for the other network. Training instances are selected based on posterior probability of each network and a selection threshold sampled from a left-tailed beta distribution. The method does not require any a-priori knowledge about the level of label noise and it can be applied to a variety of classification problems including medical tasks such as classification of medical signals and semantic segmentation of medical images.

## Data Availability

This study utilized five publicly available datasets: MNIST, CIFAR-10, CIFAR-100, Sunnybrook Cardiac Data, and the PTB-XL dataset. The MNIST^51^ database of handwritten digits can be accessed from Yann LeCun’s website (http://yann.lecun.com/exdb/mnist/). The CIFAR-10^52^ and CIFAR-100^53^ datasets are accessible from the website of the Canadian Institute For Advanced Research (https://www.cs.toronto.edu/~kriz/cifar.html). The Sunnybrook Cardiac Data^62^ for cardiac MR left ventricle segmentation can be accessed from the Cardiac Atlas Project website (http://www.cardiacatlas.org/studies/sunnybrook-cardiac-data/). The PTB-XL electrocardiography dataset^59^ is accessible from the PhysioNet website (https://physionet.org/content/ptb-xl/1.0.1/).
